# Incidence of cerebral cavernous malformations among adults in Scotland: a prospective, population-based study

**DOI:** 10.1136/jnnp-2025-338343

**Published:** 2026-04-15

**Authors:** Abel Clemens Adriaan Sandmann, Jonathan M Coutinho, Dagmar Verbaan, William Peter Vandertop, Philip Michael White, Rustam Al-Shahi Salman

**Affiliations:** 1 Department of Neurology, Amsterdam UMC, University of Amsterdam, Amsterdam, The Netherlands; 2 Neurovascular Disorders, Amsterdam Neuroscience, Amsterdam, The Netherlands; 3 Department of Neurosurgery, Amsterdam UMC, University of Amsterdam, Amsterdam, The Netherlands; 4 Translational and Clinical Research Institute, Newcastle University, Newcastle upon Tyne, UK; 5 Institute for Neuroscience and Cardiovascular Research, The University of Edinburgh, Edinburgh, UK

**Keywords:** EPIDEMIOLOGY, CEREBROVASCULAR DISEASE, CEREBROVASCULAR, Prevalence

## Abstract

**Background:**

Information about the incidence of cerebral cavernous malformations (CCM) is sparse and the effect of increasing MRI availability is uncertain. Our objective was to assess the incidence of symptomatic and incidental CCM over time.

**Methods:**

This prospective, population-based study used multiple overlapping sources of case ascertainment to identify all adults aged ≥16 years who were newly diagnosed with CCM using brain MRI or pathology in Scotland between 1 January 1999 and 31 December 2003 or 1 January 2006 and 31 December 2010 inclusive. Crude, age-stratified, sex-stratified and symptomatic versus incidental CCM incidence rates were calculated using Scottish mid-year population estimates.

**Results:**

The crude incidence for 300 newly diagnosed CCM cases (median age 44 years, 141 (47%) men) was 0.72 (95% CI 0.64 to 0.80) per 100 000 adults per year, with similar rates for men and women. The incidence of symptomatic CCM was higher than that of incidentally detected CCM (0.40 (0.34 to 0.47) versus 0.31 (0.26 to 0.37), p=0.028). Between 1999–2003 and 2006–2010, there were statistically non-significant increases in symptomatic and incidental incidence of CCM (0.36 (0.28 to 0.45) to 0.44 (0.36 to 0.54), p=0.18; 0.30 (0.23 to 0.38) to 0.33 (0.26 to 0.41), p=0.59). The estimated probability of becoming symptomatic for people with asymptomatic CCM was 0.25% (0.22%–0.29%) per year. Overall incidence increased with age, adjusted for year of diagnosis and sex (adjusted incidence rate ratio 1.09 (1.01 to 1.17) per decade increase, p=0.019).

**Conclusions:**

The incidence of symptomatic CCM exceeded that of incidentally discovered CCM; however, no significant change was observed over time. Approximately 1 in 400 people with asymptomatic CCM become symptomatic annually. These findings can help plan healthcare service delivery and research study recruitment.

**WHAT IS ALREADY KNOWN ON THIS TOPIC**

- The reported incidence of cerebral cavernous malformations ranges from 0.17 to 2.01 per 100 000 individuals per year. However, existing estimates are derived from retrospective, hospital-based studies or lack analysis of temporal changes.

**WHAT THIS STUDY ADDS**

- In this prospective, population-based study of adults newly diagnosed with cerebral cavernous malformations over 10 years, the crude incidence was 0.72 per 100 000 per year. Symptomatic incidence exceeded that of incidentally discovered cases, with no evidence of change over time.

**HOW THIS STUDY MIGHT AFFECT RESEARCH, PRACTICE OR POLICY**

- Stable incidence rates could inform healthcare service planning and indicate that adequately powered clinical studies will require multicentre collaboration.

## Introduction

 Cerebral cavernous malformations (CCM) are intracranial vascular malformations that can be diagnosed incidentally in people who are asymptomatic or have unrelated symptoms, as well as after the onset of CCM-related epileptic seizures or focal neurological deficits, either of which may be provoked by intracranial haemorrhage.^1^ Although the majority of CCMs are sporadic and solitary, around one-fifth of cases have multiple CCMs, typically due to a familial form.^2^ Although the clinical course of CCM is often relatively mild,^3–5^ a diagnosis may significantly reduce patients’ quality of life.^6 7^

Despite the clinical importance of CCM, knowledge about its epidemiology is limited. The population-based incidence of CCM has first been reported to be 0.17 (95% CI 0.00 to 0.34) per 100 000 per year between 1965 and 1992.^8^ However, this rate is likely an underestimate because all cases were diagnosed after 1985, and MRI became increasingly available only towards the end of the study. In 1999–2000, among adults in Scotland, the incidence was 0.56 (95% CI 0.41 to 0.75).^9^ The incidence of CCM varied between 1.86 and 2.01 per 100 000 people during 1995–2004 and 2004–2020, respectively, in two retrospective, hospital-based studies.^10 11^ However, these studies used estimated catchment areas of tertiary referral centres as the denominator, which may have overestimated the rates.

Currently, the effect of increasing MRI availability on CCM detection remains uncertain. Precise incidence estimates are relevant for clinical practice, as they help plan healthcare service delivery and guide the feasibility of research studies.^12^ However, truly population-based incidence data remain scarce. Therefore, this study aimed to evaluate the incidence of symptomatic and incidental CCM over time in a prospective, population-based study. We hypothesised that the incidence increased over time due to the expanding availability and growing use of MRI over the past decades.

## Methods

### Study design

The Scottish Audit of Intracranial Vascular Malformations (SAIVMs) was a National Health Service clinical audit approved by the Multicentre Research Ethics Committee for Scotland (MREC/98/0/48). SAIVMs included people if they had a first-in-a-lifetime diagnosis of any type of intracranial vascular malformation, whether symptomatic or not, made between 1 January 1999 and 31 December 2003 or 1 January 2006 and 31 December 2010, if they were ≥16 years and if they were permanent residents in Scotland.^9 13^ Diagnoses made in 2004 and 2005 were not included, as these years fell between the predefined study periods. The year of diagnosis was determined by the date of the radiological or pathological examination that established the diagnosis.

In SAIVMs, multiple overlapping sources were used to recruit a population-based cohort prospectively, which included a collaborative nationwide network of neurologists, neurosurgeons, stroke physicians, radiologists and pathologists, as well as centralised, routine coding of hospital discharge records and death certificates,^9^ using codes for vascular malformations of the brain.^13^ Owing to the limitations of the International Classification of Diseases, 10th Revision, codes specific to intracranial vascular malformations were only available for brain arteriovenous malformations (I60.8 and Q28.2).^14^ When this approach was evaluated, >97% of brain arteriovenous malformations were ascertained by the study’s methods,^13^ which provides reassurance about the completeness of ascertainment of CCM.

Each CCM diagnosis was made according to operational diagnostic criteria and allocated a measure of certainty (definite, probable or possible),^13 15^ as uncertainty may exist even with complete radiological or pathological examination. Each radiological diagnosis was discussed and verified during consensus meetings with consultant neuroradiologists using the diagnostic brain imaging studies. Reports from pathologists were used if the diagnosis was made following surgical excision or autopsy. Only definite cases among living individuals were included to determine the incidence in the living population, whereas those diagnosed at autopsy were excluded.

Data collected at baseline included demographic, clinical presentation and CCM characteristics. The mode of presentation was classified as either symptomatic or incidental. CCM characteristics included the number of CCMs, CCM location (supratentorial, infratentorial or supra- and infratentorial), presence of a brainstem CCM, temporal cortical/superficial CCM or associated developmental venous anomaly (DVA), and the largest CCM diameter in any direction. Prospective follow-up was conducted annually until SAIVMs was concluded on 31 December 2023, with a completeness of follow-up of 95%.^4^

### Demographics of Scotland

The population in Scotland is determined through decennial censuses, with the most recent conducted in March 2022, providing mid-year population estimates, stratified by age and sex. This allowed us to determine the overall, sex-stratified and age-stratified population denominator of adults aged ≥16 years in Scotland during the study years. These mid-year population estimates are provided in online supplemental figure 1.

Geographical areas in Scotland are allocated a deprivation category, which is a composite measure that reflects the socio-economic status of that area. Areas are divided into seven categories ranging from 1 (most deprived) to 7 (least deprived), with approximately equal numbers of areas in each category. The deprivation category incorporates several domains, such as income, employment, education, housing, health and access to services. Each study participant was assigned a deprivation score according to their area of residence at the time of diagnosis.

### Statistical analysis

The crude incidence was calculated using the total number of cases over the 10-year study period as the numerator and the sum of the corresponding mid-year population estimates as the denominator. We also calculated rates for the two study periods separately (1999–2003 and 2006–2010), as well as rates per year. In addition to crude incidence, rates were calculated stratified by sex and age (per decade). Prevalent people with CCM were not removed from the denominator because we considered the number likely to be so small as to make negligible effect on the incidence, and the population prevalence of all CCM has not been reliably determined. We used the χ2 test for comparisons of incidence stratified by sex, age or time.

In addition to stratifying the incidence by demographic factors, we quantified the incidence of symptomatic and incidental CCM, as well as brainstem CCM, using the overall population estimate as the denominator. We performed a secondary analysis of symptomatic detection among the number of people estimated to have asymptomatic CCM, based on the prevalence of CCM in people without neurological symptoms who underwent brain MRI (0.16% (95% CI 0.10% to 0.23%) or 1 in 625 individuals),^16^ which we assumed would remain constant over time. Therefore, we considered the prevalence of asymptomatic CCM during the study to be 160 per 100 000 people, which includes cases that have or have not (yet) been diagnosed. Subsequently, we divided the calculated rate for symptomatic incidence (per 100 000 per year) by this prevalence rate, thereby assessing the annual probability to become symptomatic.

A Poisson regression analysis of incidence was performed, including mid-year population estimates for each stratum as an offset to account for the population at risk. Independent factors that were considered for inclusion were the year of diagnosis, age, sex and deprivation category, if such population data were available for each stratum. Age (per decade) was included as a continuous variable because we did not have a hypothesis about its relationship with CCM incidence. For each factor, unadjusted and adjusted incidence rate ratios (IRR) were calculated. Analyses were performed on anonymised data extracts using IBM SPSS Statistics version 28.^17^

## Results

From 1 January 1999 to 31 December 2003 and 1 January 2006 to 31 December 2010 inclusive, 306 adult residents in Scotland were newly diagnosed with one or more definite CCM (295 on brain MRI, 5 after surgical excision and 6 at autopsy). After excluding the six cases diagnosed incidentally at autopsy that did not represent incidence in the living population, 300 cases were included. The median age was 44 (IQR 32–57) years, 141 (47%) were men and 169 (56%) were symptomatic (table 1). Brainstem CCM occurred in 48 (18%) patients, of whom 36 (75%) were symptomatic. Baseline characteristics by year of diagnosis are provided in online supplemental table 1.

**Table 1:** Baseline characteristics of people diagnosed with CCM

	Overall (n=300)	1999-2003 (n=135)	2006-2010 (n=165)
Age (years)	44 (32–57)	40 (31–53)	46 (33–60)
Sex			
Male	141 (47%)	56 (41%)	85 (52%)
Female	159 (53%)	79 (59%)	80 (48%)
Mode of presentation			
Symptomatic	169 (56%)	74 (55%)	95 (58%)
Incidental	131 (44%)	61 (45%)	70 (42%)
Multiple CCMs	57 (19%)	26 (19%)	31 (19%)
Location of CCM			
Supratentorial	215 (72%)	97 (72%)	118 (72%)
Infratentorial	54 (18%)	27 (20%)	27 (16%)
Supratentorial and infratentorial	31 (10%)	11 (8%)	20 (12%)
Brainstem CCM	48 (16%)	18 (13%)	30 (18%)
Temporal cortical/superficial CCM	55 (18%)	23 (17%)	32 (19%)
Largest CCM diameter (mm)	12 (8–18)	12 (10–18)	11 (8–17)
Associated DVA	28 (9%)	20 (15%)	8 (5%)
Deprivation category			
1	23 (8%)	12 (9%)	11 (7%)
2	51 (17%)	15 (11%)	36 (22%)
3	70 (23%)	38 (28%)	32 (19%)
4	82 (27%)	35 (26%)	47 (28%)
5	35 (12%)	16 (12%)	19 (12%)
6	22 (7%)	10 (7%)	12 (7%)
7	17 (6%)	9 (7%)	8 (5%)

Data are median (IQR) or number (%).

CCM, cerebral cavernous malformation; DVA, developmental venous anomaly.

**Figure 1:**
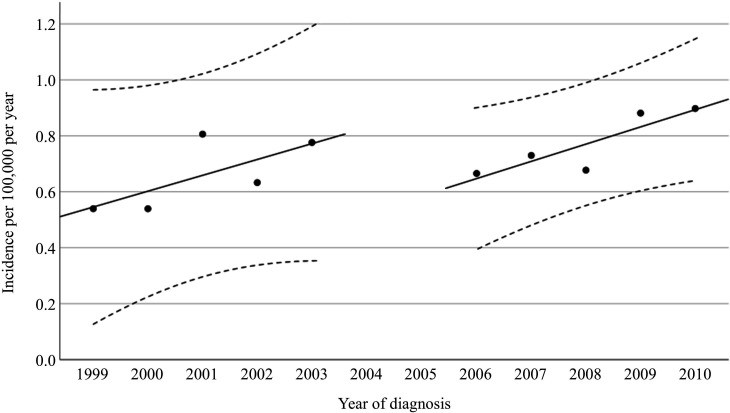
Incidence of CCM among adults (aged ≥16 years) in Scotland per year of diagnosis; the dashed lines represent the 95% CI of the estimated linear association. CCM, cerebral cavernous malformation.

During the 10-year study period, the crude incidence of CCM was 0.72 (95% CI 0.64 to 0.80) per 100 000 adults per year. Between 1999–2003 and 2006–2010, the number of cases increased from 135 to 165, which was not statistically significant considering a concomitant rise in the average mid-year adult population from 4 095 833 to 4 278 087, resulting in a non-significant increase of the crude incidence from 0.66 (95% CI 0.55 to 0.78) to 0.77 (95% CI 0.66 to 0.90, p=0.18). Figure 1 presents per-year incidence rates, demonstrating a non-significant trend towards an increasing incidence.

**Figure 2:**
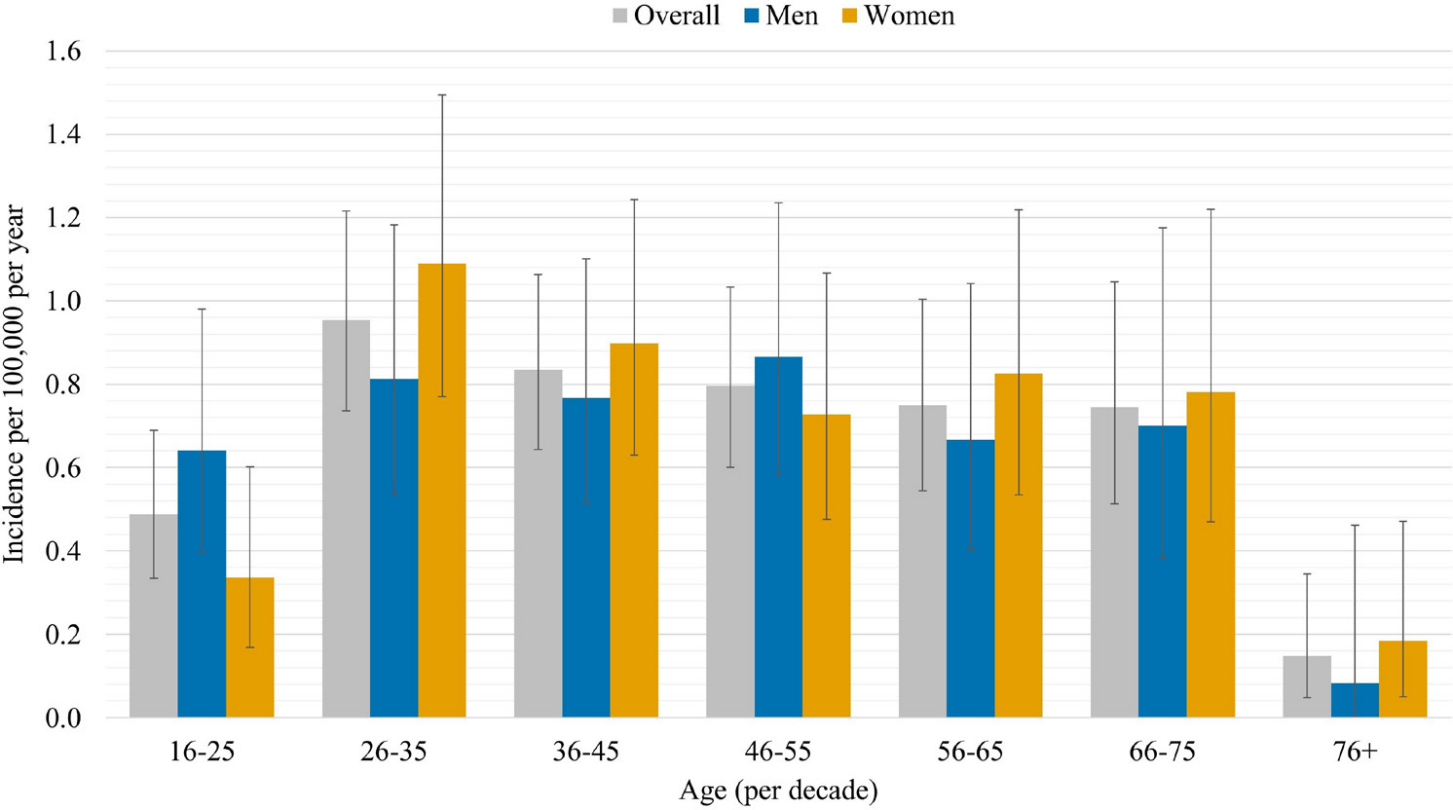
Age-stratified incidence of CCM among adults (aged ≥16 years) in Scotland. CCM, cerebral cavernous malformation.

The age-stratified incidence rates of CCM per decade are shown in figure 2 (age-stratified incidence rates by study period are presented in online supplemental table 2). The incidence of CCM stratified by sex was 0.71 (95% CI 0.60 to 0.84) per 100 000 adult males per year and 0.72 (95% CI 0.62 to 0.85) per 100 000 adult females per year (p=0.86). For men, the incidence increased between 1999–2003 and 2006–2010 from 0.58 (95% CI 0.44 to 0.75) to 0.83 (95% CI 0.67 to 1.03, p=0.033), whereas for women, there was no increase (0.73 (95% CI 0.58 to 0.91) and 0.72 (95% CI 0.57 to 0.89), respectively, p=0.88).

During the study, the incidence of symptomatic CCM was 0.40 (95% CI 0.34 to 0.47) per 100 000 adults per year, which was higher than that of incidentally detected CCM (0.31 (95% CI 0.26 to 0.37), p=0.028). Between 1999–2003 and 2006–2010, there were statistically non-significant increases for both symptomatic CCM (from 0.36 (95% CI 0.28 to 0.45) to 0.44 (95% CI 0.36 to 0.54), p=0.18) and incidentally detected CCM (from 0.30 (95% CI 0.23 to 0.38) to 0.33 (95% CI 0.26 to 0.41), p=0.59).

The incidence of brainstem CCM was 0.11 (95% CI 0.08 to 0.15) per 100 000 adults per year, with a non-significant increase between 1999–2003 and 2006–2010 (from 0.09 (95% CI 0.05 to 0.14) to 0.14 (95% CI 0.09 to 0.20), p=0.11). The incidence of symptomatic brainstem CCM was higher than that of incidentally detected brainstem CCM (0.09 (95% CI 0.06 to 0.12) vs 0.03 (95% CI 0.01 to 0.05), p<0.001).

**Figure 3:**
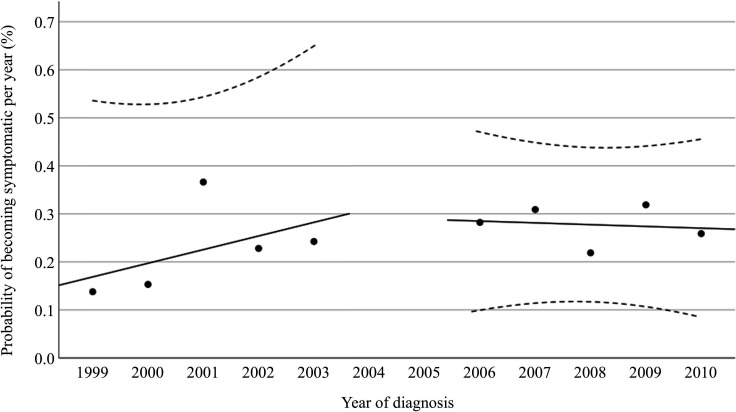
Probability of becoming symptomatic among people estimated to have asymptomatic CCM per year of diagnosis; the dashed lines represent the 95% CI of the estimated linear association. CCM, cerebral cavernous malformation.

In a secondary analysis of symptomatic detection among people with asymptomatic CCM, the estimated probability to become symptomatic was 0.25% (95% CI 0.22% to 0.29%) each year. Between 1999–2003 and 2006–2010, there was a statistically non-significant increase in the annual probability from 0.23% (95% CI 0.18% to 0.28%) to 0.28% (95% CI 0.23% to 0.34%, p=0.18). Per-year probabilities are presented in figure 3.

In Poisson regression analysis, the year of diagnosis was not associated with the incidence of CCM (table 2). When adjusted for the year of diagnosis and sex, incidence was found to be positively associated with an older age (adjusted IRR 1.09 (95% CI 1.01 to 1.17) per decade increase, p=0.019). Deprivation category could not be included because population data stratified by year, age, sex and deprivation category were incomplete for the study (only available from 2001) and expressed as deciles (ranked into 10 equal groups), whereas our study used a seven-category ordinal scale, making the two measures incompatible for comparison.

**Table 2:** Poisson regression model for associations with the incidence of CCM among adults (aged ≥16 years) in Scotland

	All patients (n=300)	Unadjusted IRR (95% CI)	P-value	Adjusted IRR (95% CI)	P-value
Year of diagnosis (1-year increments)	1999–2003 and 2006–2010	1.02 (0.99 to 1.05)	0.14	1.02 (0.99 to 1.05)	0.18
Age (per-decade increment)	44 (32–57)	1.09 (1.02 to 1.17)	**0.018**	1.09 (1.01 to 1.17)	**0.019**
Female (vs) male	159 (53%)	0.93 (0.74 to 1.17)	0.55	0.92 (0.73 to 1.15)	0.47

Data are ranges, median (IQR), number (%) or IRR (95% CI); p-values in bold were considered statistically significant (p<0.05).

CCM, cerebral cavernous malformation; IRR, incidence rate ratio.

## Discussion

In this prospective, population-based study covering over 10 years, the crude incidence of adults newly diagnosed with CCM was 0.72 (95% CI 0.64 to 0.80) per 100 000 adults per year. The incidence of symptomatic CCM exceeded that of incidentally detected CCM, particularly for brainstem CCM. The probability of becoming symptomatic among all people estimated to have asymptomatic CCM was approximately 0.25% per year. In other words, 1 in 400 such people will become symptomatic annually. Although the incidence did not increase over time, age-specific incidence increased per decade of age.

Accurate estimates of the incidence of CCM require prolonged, prospective ascertainment from a large, well-defined, stable population. The Scottish population has a manageable size to assess CCM incidence and allowed the establishment of the current prospective, population-based study. In an early publication of this study, the crude incidence of CCM during 1999–2000 was 0.56 (95% CI 0.41 to 0.75),^9^ whereas the incidence over 10 years up to 2010 (with a gap in 2004–2005) was 0.72 (95% CI 0.64 to 0.80). This finding may suggest that the incidence increased over time. However, the year of diagnosis was not significantly associated in Poisson regression analysis.

The only other truly population-based study on the incidence of CCM in the literature was a retrospective investigation of the population of Olmsted County, Minnesota (approximately 124 000 people) over 27 years, using the Mayo Clinic Medical Records Linkage System.^8^ Five CCM cases were detected, of whom one was incidentally found during autopsy, yielding an incidence of 0.17 (95% CI 0.00 to 0.34) per 100 000 per year. However, the study’s population denominator may not suffice for surveys of a condition with an incidence below 1 per 100 000 per year. Moreover, case ascertainment was based on medical records rather than prospective MRI or pathology studies, and all cases were diagnosed after 1985 (incidence of 0.50 (95% CI 0.00 to 1.00) during 1985–1992), only when increasing availability of MRI started.

Additional CCM incidence studies were retrospective and used the estimated catchment areas of tertiary referral centres where the CCM diagnosis was made as the denominator.^10 11^ A study, conducted in Bern, Switzerland, found that 347 cases (10 spinal and five orbital) were diagnosed over 20 years, but the reported symptomatic and asymptomatic rates (1.31 and 0.55 per 100 000 per year) were only based on the last 10 years of the study when the diagnosis rate ‘became relatively constant after a progressive increase’.^10^ Moreover, although the authors excluded cases from abroad, the population denominator could still not be considered as stable, as individuals from within the catchment area may have been diagnosed elsewhere, and cases diagnosed at the hospital may have been residents outside the catchment area, which was not verified.

The issue of an uncertain population denominator also accounts for a study conducted at two university hospitals in Finland that found a crude CCM incidence rate of 2.01 (95% CI 1.85 to 2.16) per 100 000 per year, which increased between 2004 and 2020.^11^ The authors compared this high rate with the incidence in Scotland during 1999–2000^9^ and partly explained the large difference by the different study periods, as MRI became more widely available over time. However, this explanation is challenged by the present study, which includes data up to 2010. The authors used the same reasoning to explain the increase in the incidence of particularly incidental CCM. We believe that the increasing availability and growing use of MRI contribute to higher reported rates of both incidental and symptomatic CCM. Wider use of MRI naturally leads to the discovery of more asymptomatic cases, whereas the expanding availability decreases the threshold for performing MRI in symptomatic patients with only minor symptoms.

This study shows that the incidence of CCM seems the lowest in the youngest and oldest age categories. The Finnish study observed the same trend and additionally reported a lower incidence of symptomatic CCM in older people.^11^ The authors suggested that most CCMs develop in middle-aged individuals and that the risk of CCM-related symptoms decreases with age. However, we believe that this pattern may reflect a bias from clinical practice, where physicians are more likely to perform MRI in middle-aged patients, particularly if symptoms are mild. Older individuals are also more likely to have relative or absolute contraindications to MRI (eg, pacemakers, other non-compatible implants, impaired renal function, cognitive impairment, movement disorders and severe cardiopulmonary disease). This interpretation is supported by the absence of literature describing biological mechanisms that would explain a higher symptomatic incidence in this age group. Therefore, we included age (in decades) as a continuous rather than an ordinal categorical variable in the Poisson regression analysis of incidence, which revealed an association with older age, although the association may not be linear.

Several studies have investigated the prevalence of CCM. In a second population-based study from Olmsted County, Minnesota, the prevalence of either symptomatic or incidental CCM among adults aged 50–89 years was 0.46% (95% CI 0.05% to 0.86%).^18^ All participants were enrolled over 12 years and underwent brain MRI; however, gradient recalled echo sequences were only added to the protocol in the last 5 years. Additional studies assessed the frequency of CCM in MRI studies of asymptomatic people and were synthesised in two systematic reviews with meta-analyses.^16 19^ The most recent review of nearly 35 000 people (including people with headache or other conditions) found a prevalence of 0.39% (95% CI 0.23% to 0.58%).^19^ In the secondary analysis of symptomatic detection among people with asymptomatic CCM, we used the other review that only included people without neurological symptoms (n=15 000, prevalence 0.16% (95% CI 0.10% to 0.23%)).^16^ Nevertheless, the prevalence of asymptomatic CCM at any moment in time remains indeterminable because of the possibly dynamic development and de novo appearance of CCMs.

We estimated that the probability of becoming symptomatic among people with asymptomatic CCM was 0.25% (95% CI 0.22% to 0.29%) per year. Although this rate and its 95% CI were calculated from point estimates, potentially underestimating the variability, the modest probability could qualify symptomatic CCM as a rare condition.^18^ This has implications for healthcare service planning and power calculations in future trials. This is supported by a recent pilot trial that compared CCM intervention (microsurgical resection or stereotactic radiosurgery) with conservative management for symptomatic CCM, which calculated that 590–1900 participants might be needed to reach a sample size required for the evaluation of treatment effects,^12^ underscoring the need for multicentre, likely even international, involvement.

A strength of this study is its population-based design, which involved prospective case identification and multiple overlapping sources of ascertainment. In contrast, hospital-based studies or those based on disease-coding alone are subject to selection and referral biases, as they may miss people who do not warrant hospital admission (eg, asymptomatic people or those unsuitable for treatment) and represent local referral practices and treatment preferences. Moreover, this study spanned 10 years, which enabled analyses over time. The use of revised population data from 2022 that corrected errors in the 2011 census ensured accurate denominator estimates. Furthermore, cases diagnosed at autopsy were excluded to reflect the clinically more relevant incidence of CCM in the living population and because autopsy rates are declining worldwide.^20 21^

Some limitations should also be considered. First, the lack of deprivation data suitable for comparison prevented accounting for its potential influence on the incidence (eg, access to healthcare and diagnostic imaging). However, any deprivation impact may be limited in Scotland, given the universal access to the public healthcare system and because CCM is biologically unlikely to be influenced by socio-economic status. Second, data on the number of MRI studies or scanners in Scotland during the study years were lacking, which prevented the assessment of incidence independent of variation in MRI availability and use. Third, data on the MRI sequences used to establish each diagnosis were unavailable, whereas with the use of susceptibility-weighted imaging and other hemosiderin-sensitive sequences, the incidence of CCM is expected to rise.

This study reports crude, sex-stratified and age-stratified incidence of CCM and found that the incidence of symptomatic CCM exceeds that of incidentally discovered CCM. Meanwhile, for people with asymptomatic CCM, the annual probability to become symptomatic is low. Although the incidence seemed to increase over time, particularly for symptomatic CCM, which may largely reflect a lowered threshold for performing brain MRI if symptoms are mild, the year of diagnosis was not associated with incidence. The study period coincided with a time period when the use of MRI was already widespread in clinical practice, suggesting that the findings reflect contemporary diagnostic practices. The presented incidence rates may help plan healthcare service delivery and research study recruitment, which will require international, multicentre involvement.

## Supplementary material

10.1136/jnnp-2025-338343 online supplemental file 1

## Data Availability

Data are available upon reasonable request.
