# Supplementary material for: Incidence of cerebral cavernous malformations among adults in Scotland: a prospective, population-based study
Source: J Neurol Neurosurg Psychiatry. 2026 Apr 15;97(9):e338343. doi: 10.1136/jnnp-2025-338343 (PMC13479685; doi:10.1136/jnnp-2025-338343)
Supplement: Supplementary file 1 [file jnnp-97-9-s001.docx]

**Supplementary Material**

Supplement to: *Incidence of cerebral cavernous malformations among adults in Scotland: a prospective, population-based study*

Supplementary Figure 1. Total and sex-stratified mid-year population estimates of adults (16 years or older) in Scotland per SAIVMs year

M, million; SAIVMs, Scottish Audit of Intracranial Vascular Malformations.

Supplementary Table 1. Baseline characteristics of people diagnosed with CCM per year of diagnosis

| **All patients** | **Age (years)** | **Sex** | | **Mode of presentation** | | **Multiple CCMs** | **Brainstem CCM** | **Temporal cortical/ superficial CCM** | **Largest CCM diameter (mm)** |
| --- | --- | --- | --- | --- | --- | --- | --- | --- | --- |
|  |  | **Male** | **Female** | **Symptoms** | **Incidental** |  |  |  |  |
| Overall (n=300) | 44 (32-57) | 141 (47%) | 159 (53%) | 169 (56%) | 131 (44%) | 57 (19%) | 48 (16%) | 55 (18%) | 12 (8-18) |
| **Per cohort** | **Age (years)** | **Sex** | | **Mode of presentation** | | **Multiple CCMs** | **Brainstem CCM** | **Temporal cortical/ superficial CCM** | **Largest CCM diameter (mm)** |
|  |  | **Male** | **Female** | **Symptoms** | **Incidental** |  |  |  |  |
| 1999-2003 (n=135) | 40 (31-53) | 56 (41%) | 79 (59%) | 74 (55%) | 61 (45%) | 26 (19%) | 18 (13%) | 23 (17%) | 12 (10-18) |
| 2006-2010 (n=165) | 46 (33-60) | 85 (52%) | 80 (48%) | 95 (58%) | 70 (42%) | 31 (19%) | 30 (18%) | 32 (19%) | 11 (8-17) |
| **Per year** | **Age (years)** | **Sex** | | **Mode of presentation** | | **Multiple CCMs** | **Brainstem CCM** | **Temporal cortical/ superficial CCM** | **Largest CCM diameter (mm)** |
|  |  | **Male** | **Female** | **Symptoms** | **Incidental** |  |  |  |  |
| 1999 (n=22) | 42 (34-54) | 10 (45%) | 12 (55%) | 9 (41%) | 13 (59%) | 4 (18%) | 6 (27%) | 5 (23%) | 11 (10-15) |
| 2000 (n=22) | 38 (32-50) | 8 (36%) | 14 (64%) | 10 (45%) | 12 (55%) | 4 (18%) | 1 (5%) | 5 (23%) | 10 (8-15) |
| 2001 (n=33) | 39 (31-49) | 14 (42%) | 19 (58%) | 24 (73%) | 9 (27%) | 6 (18%) | 5 (15%) | 5 (15%) | 12 (10-20) |
| 2002 (n=26) | 34 (26-55) | 10 (38%) | 16 (62%) | 15 (58%) | 11 (42%) | 5 (19%) | 1 (4%) | 7 (27%) | 15 (10-20) |
| 2003 (n=32) | 46 (36-60) | 14 (44%) | 18 (56%) | 16 (50%) | 16 (50%) | 7 (22%) | 5 (16%) | 1 (3%) | 12 (8-15) |
| 2006 (n=28) | 46 (37-63) | 13 (46%) | 15 (54%) | 19 (68%) | 9 (32%) | 6 (21%) | 5 (18%) | 10 (36%) | 15 (8-19) |
| 2007 (n=31) | 42 (29-56) | 13 (42%) | 18 (58%) | 21 (68%) | 10 (32%) | 8 (26%) | 5 (16%) | 4 (13%) | 12 (9-19) |
| 2008 (n=29) | 49 (34-62) | 18 (62%) | 11 (38%) | 15 (52%) | 14 (48%) | 3 (10%) | 7 (24%) | 4 (14%) | 9 (7-15) |
| 2009 (n=38) | 50 (39-60) | 17 (45%) | 21 (55%) | 22 (58%) | 16 (42%) | 6 (16%) | 6 (16%) | 9 (24%) | 12 (8-18) |
| 2010 (n=39) | 42 (31-60) | 24 (62%) | 15 (38%) | 18 (46%) | 21 (54%) | 8 (21%) | 7 (18%) | 5 (13%) | 10 (8-13) |

Data are median (IQR) or number (%); CCM, cerebral cavernous malformation; IQR, interquartile range.

Supplementary Table 2. Age-stratified incidence of CCM among adults (16 years or older) in Scotland per study period

|  | **Overall** | | **1999-2003** | | **2006-2010** | |
| --- | --- | --- | --- | --- | --- | --- |
|  | **N** | **Incidence** | **N** | **Incidence** | **N** | **Incidence** |
| **Overall** | 300 | 0.72 (0.64-0.80) | 135 | 0.66 (0.55-0.78) | 165 | 0.77 (0.66-0.90) |
| **Age** |  | | | | | |
| **16-25** | 32 | 0.49 (0.33-0.69) | 15 | 0.48 (0.27-0.79) | 17 | 0.50 (0.29-0.80) |
| **26-35** | 65 | 0.95 (0.74-1.22) | 35 | 0.98 (0.68-1.36) | 30 | 0.93 (0.63-1.33) |
| **36-45** | 65 | 0.83 (0.64-1.06) | 31 | 0.80 (0.55-1.14) | 34 | 0.86 (0.60-1.21) |
| **46-55** | 56 | 0.80 (0.60-1.03) | 29 | 0.85 (0.57-1.23) | 27 | 0.74 (0.49-1.07) |
| **56-65** | 44 | 0.75 (0.54-1.01) | 10 | 0.37 (0.18-0.67) | 34 | 1.08 (0.75-1.51) |
| **66-75** | 33 | 0.74 (0.51-1.05) | 13 | 0.60 (0.32-1.03) | 20 | 0.88 (0.54-1.37) |
| **76+** | 5 | 0.15 (0.05-0.35) | 2 | 0.12 (0.02-0.45) | 3 | 0.17 (0.04-0.50) |

Data are number or rate (95% CI) per 100,000 adults per year; CCM, cerebral cavernous malformation; CI, confidence interval.
